# Inhalation of Chlorine in Phthisis

**Published:** 1831

**Authors:** 


### XXXIX.

### INHALATION OF CHLORINE IN PHTHISIS.

IN a late number of the Archives Genérales, M. Cottereau, has detailed thirteen cases, where inhalation proved curative or beneficial in cases of phthisis, or diseases very much resembling phthisis. We shall glance at a few of these, while waiting for the statement of failures that is promised by the author.

*Case* 2. Was a lady the wife of a medical man, delicate and nervous, who in the year 1828 became affected with cough, and pain in the chest. Her husband applied leeches but with only temporary relief. Numerous blisters followed, with different medicines, all unsuccessfully. A consultation was called; the expectoration was found to be purulent, and the fever hectic. Emaciation advanced rapidly. The stethoscope now discovered pectoriloquy at the summit of the right lung. In the other lung there was a dull sound for a considerable extent. Five days afterwards, the patient was examined by another physician, who came to the same auscultic conclusions, and pronounced the disease to be phthisis unequivocally. The inhalation of chlorine gas was then determined on. We need not detail the proceedings. The process was commenced on the 15th of May, by eight inhalations, with three drops of the solution in each. On the 18th of June, the cure was pronounced as complete.

We confess we are very far from being convinced that the above was a case of real phthisis, notwithstanding the ausculti indications which are laid down on paper; but which we have great reason to distrust, from the innumerable errors which we every day see committed in practice. We shall, however, give one more case, because it is the one which Dr. Cottereau considers as the most unequivocal.

*Case* 12. Madam N. aged 27 years, of delicate constitution, lymphatic temperament, and phthisical family, was rickety in her infancy, and afterwards scrofulous. In December, 1827, she was seized with a dry cough after an abortion. The cough and other pulmonary symptoms increased, and in April 1828, she again became pregnant, and the pectoral phenomena instead of being mitigated, were exasperated, and accompanied by hæmoptysis. Nine months after that she had a safe delivery, after which the pectoral symptoms increased in severity. In July 1829, when M. Cottereau first saw her, she was greatly emaciated, the skin had a dry leaden aspect, the eyes were dull, the extremities frequently cold, the appetite indifferent, her cough not severe but frequent, especially in the morning, with copious easy expectoration of greenish yellow or greyish, opaque, consistent masses, amidst clear, viscous mucosity; there was also constant pain in the larynx and between the shoulders, with shifting pains throughout the chest,—great oppression,—and almost complete loss of voice. The sound on percussion was very clear in a circumscribed spot about an inch under the right clavicle; around this spot perfectly dull, and throughout the rest of the right side somewhat obscure; but on the left side it was natural, except that some obscurity of sound existed in the lower third. In the axilla, as well as in the right subclavicular region, at the spot already mentioned, there was unequivocal cavernous respiration, cavernous râle and pectoriloquy —around this spot a complete absence of respiratory murmur, and in the rest of the right lung tracheal respiration with slight crepitating râle, — in the left side natural respiration in the two upper thirds, mucous râle in the lower third. The pulse was 80, but very irritable under the slightest exercise, the breathing always hurried, and a hectic paroxysm occurred every evening, with its usual accompaniments and termination. On the 20th July the inhalation of chlorine was begun, but after a day or two an intermission was necessary, on account of an uneasy sense of heat and dryness in the back of the throat, which appears a very common effect of the chlorine when first used. In a few days, however, it was resumed, and gradually increased to ten inhalations per day. During the three first weeks of August two other intermissions were required on account of an increase of pain and a sense of heat in the chest. About the middle of September the general symptoms were greatly mitigated. For the ensuing month the inhalation could not be persevered in, on account of the state of the chest; but it was resumed at the middle of October, and continued regularly afterwards. At the middle of November the amendment was so great that the lady considered herself quite cured. Towards the close of December her state was as follows:—The appetite, strength, and flesh natural; the cough and expectoration gone; the breathing not affected by walking or climbing; the sound on percussion dull over a circumscribed spot, corresponding with the point where pectoriloquy was formerly heard, and the respiratory râle inaudible in the same quarter; but every where else the lungs appeared perfectly in their natural condition, free of morbid râle, as well as of pectoriloquy. The inhalation was persevered in for security's sake till the middle of January.—For three months afterwards this patient continued to enjoy uninterrupted health. Subsequently, however, she was exposed to frequent fatigue and night watching, in consequence of the illness of her infant; and at the end of August she was seized with symptoms of general fever, which proved fatal in four weeks, without having ever been accompanied with any signs of an affection of the lungs. In giving an account of the dissection, we shall confine ourselves to the appearances in the respiratory organs. The epiglottis, larynx, and windpipe were natural. Two cervical glands on the right side were enlarged to the size of small nuts, and contained each a nucleus of friable, chalky-like matter. Both lungs were pale-grey, pliant, and crepitating. The left adhered here and there to the costal pleura by old adhesions. In the middle part of its upper lobe there was a tubercle as big as a pea, similar to the nuclei of the diseased cervical glands, and also some minute tubercles in the upper portion of the same lobe. The right lung was free of adhesion. "At the fore-part of its apex there was, over a space 18 lines long and 8 deep, a darker tint of the tissue, with very firm consistence, and an appearance of wrinkling; and when this mass was cut into, it was found composed of hard, compact, almost fibrous tissue, of a slate colour marbled with greyish-white; it was not traversed by any bronchial tubes, all of which were obliterated as they approached it. On the edge of this apparent cicatrix there was a small steatomatous-like tubercle, scarcely a line in diameter; and in various parts of the upper lobe of the same lung about a dozen minute miliary tubercles; but the tissue of that lung, both around the tubercles and in its other lobes, was quite healthy.

The foregoing is certainly a very strong case, *if all the particulars are candidly and honestly stated*—which, for the honour of science, we hope they are. But it is to be recollected that tubercular cavities have been known to cicatrize under other modes of treatment, and even where no treatment was ascertained. The *post hoc, ergo propter hoc* reflection, therefore, is recalled to our memory, independently of the unavoidable suspicions which attach to histories of the effects of new remedies. We shall look forward to the sequel of Dr. Cottereau's paper, where the unsuccessful cases are to be given. In the mean time, as he assures us that even in these last, there was mitigation of the symptoms, perhaps prolongation of life produced by the chlorine inhalation, we deem it a duty to throw out these faint hopes for the encouragement of our brethren.

